# Intelligent Stroke Disease Prediction Model Using Deep Learning Approaches

**DOI:** 10.1155/2024/4523388

**Published:** 2024-05-23

**Authors:** Chunhua Gao, Hui Wang

**Affiliations:** ^1^School of Tourism and Physical Health, Hezhou University, Hezhou 542899, China; ^2^School of Artificial Intelligence, Hezhou University, Hezhou 542899, China

## Abstract

Stroke is a high morbidity and mortality disease that poses a serious threat to people's health. Early recognition of the various warning signs of stroke is necessary so that timely clinical intervention can help reduce the severity of stroke. Deep neural networks have powerful feature representation capabilities and can automatically learn discriminant features from large amounts of data. This paper uses a range of physiological characteristic parameters and collaborates with deep neural networks, such as the Wasserstein generative adversarial networks with gradient penalty and regression network, to construct a stroke prediction model. Firstly, to address the problem of imbalance between positive and negative samples in the stroke public data set, we performed positive sample data augmentation and utilized WGAN-GP to generate stroke data with high fidelity and used it for the training of the prediction network model. Then, the relationship between observable physiological characteristic parameters and the predicted risk of suffering a stroke was modeled as a nonlinear mapping transformation, and a stroke prediction model based on a deep regression network was designed. Finally, the proposed method is compared with commonly used machine learning-based classification algorithms such as decision tree, random forest, support vector machine, and artificial neural networks. The prediction results of the proposed method are optimal in the comprehensive measurement index *F*. Further ablation experiments also show that the designed prediction model has certain robustness and can effectively predict stroke diseases.

## 1. Introduction

According to the World Health Organization (WHO), stroke is the second leading cause of death worldwide, accounting for about 11% of all deaths [1]. There are two major categories of risk factors for stroke, those that can be interfered with and those that cannot. Noninterventionist factors include age, gender, and genetics, while interventionist factors include hypertension, diabetes, heart disease, unreasonable diet, and nutrition. Effective assessment of the risk of stroke in high-risk groups has very positive implications for both the prevention and treatment of stroke. With the development of artificial intelligence technology, more and more scholars are using deep learning methods in the field of disease prediction, which helps in the early detection of potential disease risks for timely intervention and treatment.

Back in 1991, Wolf et al. [2] developed a health risk appraisal function for predicting stroke based on stroke risk factors including age, systolic blood pressure, diabetes, smoking, and heart disease. Wu et al. [3] established a nomogram model for the risk of stroke patients using predictors such as demographic characteristics, vascular risk factors, emotional factors, and lifestyle behaviors. Chun et al. [4] compared the utility of Framingham versus novel risk scores for the prediction of total stroke and stroke types in Chinese adults. Kent et al. [5] studied the risk of future stroke in patients with incidentally discovered silent cerebrovascular disease and identification of silent brain infarction and white matter disease from neuroimage reports through natural language processing. It can be seen that the stroke risk is predictable, but the accuracy of the prediction needs to be improved.

In recent years, machine learning algorithms have shown promising potential for stroke prediction. Zheng et al. [6] employed six machine learning methods to predict the risk of stroke, the best predictions were obtained using random forest for experimental from 233 patients, and they found that cerebral infarction, PM 8, and drinking are independent risk factors for stroke. Ma et al. [7] researched multiobjective learning and explanation for stroke risk assessment and adopted the quadratic interactive deep model to solve the problem of sample imbalance to improve the prediction accuracy. Chen and Sawam [8] investigated the use of wearable devices to monitor risk factors for stroke and analyzed the trend of combining wearable devices and machine learning algorithms to build stroke risk prediction systems. Ismail and Materwala [9] analyzed stroke data under an intelligent stroke prediction framework and compared five common machine learning algorithms: decision tree, random forest, support vector machine, naive Bayes, and logistic regression; and random forest gave the best results on the stroke test data set. Tazin et al. [10] studied the stroke prediction algorithm and utilized collected physiological data to produce datasets and used them for training machine learning algorithms such as logistic regression, decision tree, random forest, and voting classifier, with random forest predictive accuracy being the highest at nearly 96%. The use of machine learning algorithms can lead to more accurate predictions, but traditional machine learning methods usually require human involvement in the design of data features. Higher accuracy predictions can be obtained using machine learning algorithms, but traditional machine learning methods usually require human involvement in the design of data features. Deep neural networks train network models by constructing sets of positive and negative samples and automatically learn data features from the training samples, and the novel research field successfully combines machine learning and swarm intelligence approaches and proved to be able to obtain outstanding results in different areas [11–13]. The application of deep learning methods in stroke prediction has also received increasing attention [14–16], but due to the serious problem of sample imbalance in stroke dataset, the output results of deep neural network are biased. Data enhancement is a common way to deal with sample imbalance, e.g., Li et al. [17] used extremely imbalanced data augmentation generative adversarial nets to address the extremely imbalanced data augmentation problem. Mizuho et al. [18] used a single generative adversarial nets model to generate 3D CT images of lung nodules of different sizes. Hitesh et al. [19] applied various generative adversarial net-based models for data augmentation to synthesize benign and malignant mediastinal lymph node images. Han et al. [20] focused on generating synthetic multisequence brain magnetic resonance images using generative adversarial nets. Mizuho et al. [21] used artificial dataset generated by the generative adversarial network to build pretrained models for the development and evaluation of lung cancer segmentation. The above generative adversarial network-based medical image data enhancement methods have achieved better results for specific problems, stroke data is one dimensional, and its data enhancement methods need further research.

In this paper, we investigate a deep neural network-based stroke prediction system using a publicly available data set of stroke to automatically output the prediction results in an end-to-end manner. To fully exploit the potential of deep learning models, it is important to acquire large data sets. Due to the serious imbalance between positive samples (stroke patients) and negative samples (nonstroke patients) in the open stroke data set, we first used the Wasserstein generative adversarial networks with gradient penalty (WGAN-GP) [22] for positive case sample data augmentation. Then, a deep regression network model was designed for prediction based on analyzing the characteristics of the stroke observation data. In this paper, the stroke prediction problem is modeled as a regression model, and the predicted value is directly output according to the input observations, which can better assist doctors in diagnosis and effectively deal with the problem of selecting the optimal threshold of the classification model. The major contributions of this work are summarized as follows.

1. A WGAN-GP-based data enhancement method for positive case samples of stroke is proposed to generate positive case data with high fidelity and to solve the problem of serious imbalance between positive and negative case samples in the data set
2. The relationship between input observations and stroke prediction results is modeled as a problem of solving a nonlinear mapping function, and a specific solution method is given
3. The designed deep regression model performs stroke prediction without human intervention and automatically outputs stroke risk prediction results in an end-to-end manner

The remaining part of this paper is organized as follows.  Section 2 describes the stroke data set, and a detailed analysis of the stroke prediction network model was performed.  Section 3 describes the details of the experimental results, and the designed regression model is compared with typical machine learning methods. In Section 4, the experimental results are further discussed. In Section 5, the concluding remarks and future work are discussed.

## 2. Materials and Methods

### 2.1. Stroke Dataset Preprocessing

The data in this paper comes from the public data set of stroke on the Kaggle data platform [23], including 10 attribute characteristics such as heart disease, hypertension, average blood glucose, and BMI. Due to the serious imbalance in the number of samples with stroke disease (positive cases) and those without stroke disease (negative cases) in the data set, with only seven hundred or so positive cases accounting for about 2% of the samples, it is necessary to augment the positive case samples in order to improve the accuracy of the prediction model. For this, we adopt a two-stage data augmentation strategy. Firstly, each attribute feature is quantized as shown in Table 1, and the samples with missing attribute features of the positive examples are filled in with maximum probability, i.e., when the attribute features of a sample are unknown the quantized value of the feature is taken to be the one with the highest probability of occurrence among the attribute parameters (e.g., when smoking_status is unknown, the quantized value is taken to be the one with the highest probability of occurrence of the parameters never smoked, formerly smoked, and smokes in the positive example samples).

Then, WGAN-GP was used to augment the positive example samples (which will be analyzed in detail in Section 2.2), and the negative example samples were selected for the training of the prediction network model in a ratio of 1 : 1 to improve the generalization of the prediction model. The main bases for selecting the negative example samples were as follows: (1) selecting samples with defined attribute characteristics; (2) sampling the attribute characteristic categories evenly.

### 2.2. Stroke Data Enhancement Network Based on WGAN-GP

**Figure 1:**
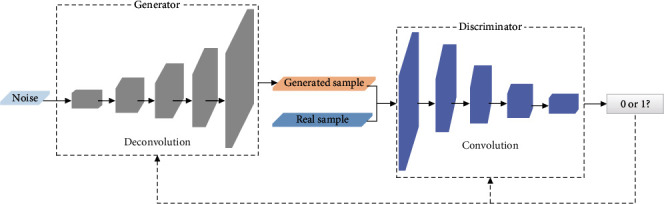
The network structure of WGAN-GP.

WGAN-GP uses the Wasserstein distance to measure the distance between the generator and the discriminator and introduces a gradient penalty term in the loss function of the discriminator to constrain the gradient paradigm of the discriminator, avoiding the problems of gradient explosion and vanishing and improving the stability of the GAN. The corresponding network structure of WGAN-GP is shown in Figure 1.

The sample data enhancement method based on generative adversarial network firstly inputs random noise and real sample data into the generative network and adversarial network of WGAN-GP, respectively, and conducts adversarial training on the generative model and discriminant model at the same time. After model training is completed, the generative model is taken out separately, and the random noise is input into the generative model to generate new sample data. As shown in the figure, the input of the generator is random noise, and the input of the discriminator is the real sample and the generated fake sample. The real sample matrix, denoted as **T** = (**C**_1_, **C**_2_, ⋯, **C**_*N*_), consists of columns C_*i*_ = (x_1,*i*_, x_2,*i*_, ⋯,x_*M*,*i*_)^T^, i.e., each real sample is composed of *N* samples randomly selected from the positive data. The parameters of the generator and discriminator network are inversely adjusted based on the output of the discriminator to generate positive example samples with high fidelity. The corresponding loss function for WGAN-GP can be expressed as follows:

(1) $$\begin{array}{c} \begin{array}{c} \text{L}=\underset{G}{\min}\;\underset{D}{\max}\left\{{\text{E}}_{\tilde{x}\sim {\text{P}}_{g}\left(\tilde{x}\right)}\left[\text{D}\left(\tilde{x}\right)\right]‐{\text{E}}_{x\sim {\text{P}}_{data}\left(x\right)}\left[\text{D}\left(\text{G}\left(x\right)\right)\right]+\lambda {\text{E}}_{\hat{x}\sim {\text{P}}_{\hat{\text{x}}}}\left[{\left({\left\|{\tilde{\text{N}}}_{\overset{\wedge }{x}}\text{D}\left(\overset{\wedge }{x}\right)\right\|}_{2}‐1\right)}^{2}\right]\right\},\\ \hat{x}=\varepsilon \tilde{x}+\left(1‐\varepsilon \right)x\;0<\varepsilon <1, \end{array} \end{array}$$

where P_g_ denotes the distribution of the generated fake sample, P_*data*_ denotes the distribution of the real sample, *λ* denotes the gradient penalty factor, which is usually taken as an empirical value (we take *λ* = 9), $\tilde{x}$ denotes the generated fake sample, $\hat{x}$ denotes a random distribution between P_*data*_ and P_*g*_, *ε* is a constant (0 < *ε* < 1). WGAN-GP is to add the regular term (gradient penalty) to the loss function of GAN in order to constrain the gradient range and solve the problems of gradient vanishing and gradient explosion. The Lipschitz limit is to require that the gradient of the discriminator does not exceed the clipping threshold, and the gradient penalty is to set an additional loss term to realize the connection between the gradient and clipping threshold. In this paper, in order to effectively deal with gradient instability, the stochastic gradient descent method (SGD) is used to optimize equation (1), and the core idea of the SGD algorithm is to randomly select a sample at each iteration to compute the gradient of the cost function and then update the parameters of the model based on this gradient. Instead of traversing the entire data set, this method uses one sample per iteration and can, therefore, significantly speed up training.

### 2.3. Stroke Prediction Network Model

**Figure 2:**
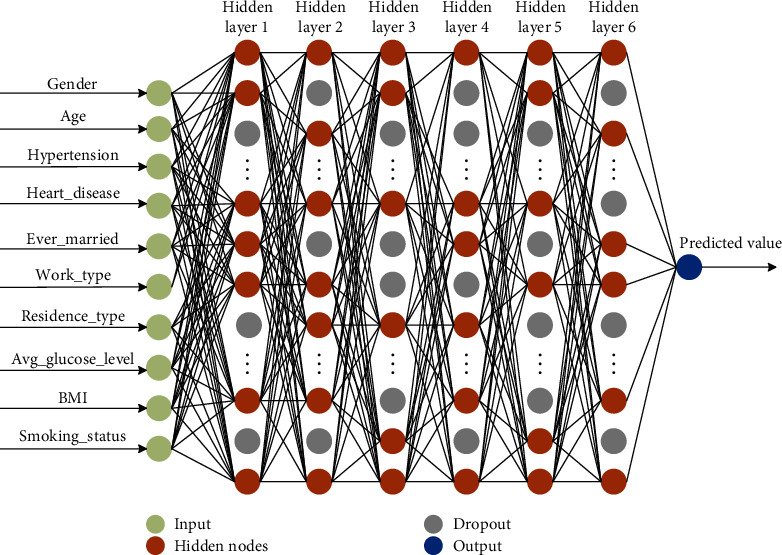
The structure of the proposed regression network.

This paper investigates the intrinsic relationship between stroke and the 10 observations (denoted as *x*_1_, *x*_2_, *x*_3_, ⋯, *x*_10_) shown in Table 1. Due to the complex etiology and pathogenesis of stroke, the determination of the outcome *y* and the input observation *x*_1_, *x*_2_, *x*_3_, ⋯, *x*_10_ usually exhibit a complex nonlinear relationship. The process of inverting *y* based on *x*_1_, *x*_2_, *x*_3_, ⋯, *x*_10_ can be described as follows:

(2) $$\begin{array}{c} y={Η}^{-1}\left\{x_{1},x_{1},x_{1},\cdots ,x_{10}\right\}, \end{array}$$

where *Η*^−1^ denotes the inverse operation and *H*^−1^(·) cannot be expressed as an explicit function. While in the mathematical point of view, in the field of artificial neural networks, the general approximation theorem [24] can be described as follows: when the number of hidden units in a feed-forward neural network is sufficiently large, the network is able to approximate the function that needs to be learned with arbitrary accuracy. This theorem shows that simple neural network architectures can be used to fit complex functions if given enough parameters. Based on this, a deep neural network can be used to characterize the intrinsic nonlinear relationship between the observed variable **X** and the predicted outcome **Y**: $Y=\hat{f}\left(X,\hat{W}\right)$, where $\hat{W}$ is the weight parameter set of the designed DNN. That is, in the expression form, a DNN is a nonlinear mapping from the observation space *𝒳* to the feature space *𝓎*, for any *x* ∈ *𝒳*, DNN(*x*) ∈ *𝓎* can be explicitly expressed as follows:

(3) $$\begin{array}{c} \text{DNN}\left(x\right)={\hat{f}}_{L}\left(\cdots {\hat{f}}_{i}\left(\cdots {\hat{f}}_{1}\left(x,w_{1}\right)\cdots ,x,w_{i}\right)\cdots ,x,w_{L}\right), \end{array}$$

where *w*_*i*_ denotes the weight coefficient (which can be determined by learning) of the *i*th layer of the DNN network. *L* denotes the depth of the DNN (which can be determined using cross-validation). To invert equation (2), *x*_1_, *x*_2_, *x*_3_, ⋯, *x*_10_ is used as input to the DNN to train the network model to output the estimation. The structure of the designed regression network is shown in Figure 2.

The detailed parameters of each layer of the regression model are shown in Table 2.

The process of stroke prediction based on the regression model can be summarized as follows:

1. Make a data set *𝒟* (with positive and negative example samples) for training DNNs:

(4) $$\begin{array}{c} D=\left\{y\left|\left(x_{1}^{\left(1\right)},x_{2}^{\left(1\right)},\cdots ,x_{10}^{\left(1\right)}\right),\cdots ,y\right.\left|\left(x_{1}^{\left(i\right)},x_{2}^{\left(i\right)},\cdots ,x_{10}^{\left(i\right)}\right),\cdots ,y\right.\left|\left(x_{1}^{\left(L\right)},x_{2}^{\left(L\right)},\cdots ,x_{10}^{\left(L\right)}\right)\right.\right\}, \end{array}$$

where *L* denotes the number of samples in the training set, *x*_1_^(*i*)^, *x*_2_^(*i*)^, ⋯, *x*_10_^(*i*)^ denotes the number of observations (including gender, age, hypertension, and heart_disease) for the *i*th sample, respectively.

(2) The nonlinear mapping process $Y=\hat{f}\left(X,\hat{W}\right)$ is unfolded into a K-layer DNN, and the network model is trained on the example set *𝒟*. In general, the results of model training can be evaluated using empirical risk, which is a measure of how well the model fits the training set in an average sense, and here, empirical risk can be defined as follows:

(5) $$\begin{array}{c} R_{emf}\left(w\right)=\frac{1}{N}\overset{N}{\underset{n=1}{\sum }}l\left(y_{n},DNN\left(x_{n};w\right)\right), \end{array}$$

where *w* denotes the set of all weighted parameter compositions in the K-layer DNN *w* = {*w*^(1)^, *w*^(2)^, ⋯, *w*^(*K*)^}, with metric function *l*(·) = ‖·‖_2_^2^. The training process of DNN is to solve the following optimization problems:

(6) $$\begin{array}{c} w^{*}=\underset{w}{\arg\min}R_{emf}\left(w\right). \end{array}$$

The gradient descent method can be used to solve this optimization problem. The details are as follows:

(3) For the observed parameter *x* ∈ *𝒳* of the input DNN, the output value *D*ΝΝ(*x*) is the stroke prediction result by nonlinear mapping transformation

**Step 1 .**

Initialization parameters. Choose an initial parameter *w*_0_ as the starting point.

**Step 2 .**

Calculate the gradient. Compute the gradient of the objective function with respect to *w*_0_ to obtain a gradient vector.

**Step 3 .**

Update parameters. Update the parameter vector in the opposite direction of the gradient according to the set learning rate.

**Step 4 .**

Repeat steps 2 and 3. Perform steps 2 and 3 iteratively until a set number of iterations is reached or the gradient change is very small.

## 3. Results

### 3.1. Data Augmentation

The unbalanced sample enhancement algorithm based on WGAN-GP in this paper is compared with the traditional synthetic minority oversampling technique (SMOTE) algorithm [25]. The GAN network utilizes the game of generative and adversarial networks to form new samples with labels, while the SMOTE algorithm interpolates the feature vectors in the feature space to form new samples by sampling. The main steps of the SMOTE algorithm are as follows:

**Step 5 .**

Calculate the distance between each minority sample and all other minority samples and find its *K* nearest neighbors (in this paper, we take *k* = 5).

**Step 6 .**

Randomly select a sample from the *K* nearest neighbors and calculate the difference between the sample and the current sample.

**Step 7 .**

According to the difference ratio, a new synthetic sample is generated, which is located on the line between the two samples.

**Step 8 .**

Repeat the above steps to generate the specified number of synthetic samples.

In order to objectively evaluate the effectiveness of dealing with sample imbalance, the WGAN-GP algorithm was compared with the WGAN algorithm and the SMOTE algorithm, and we use TPR and FPR for quantitative evaluation.

(7) $$\begin{array}{c} \begin{array}{c} \text{TPR}=\frac{\text{TP}}{\text{TP}+\text{FN}},\\ \text{FPR}=\frac{\text{FP}}{\text{FP}+\text{TN}}, \end{array} \end{array}$$

where TP denotes true positives, FN denotes false negatives, FP represent false positives, TN denotes true negatives. The results of TPR and FPR for 4250 sets of enhanced data are shown in Table 3.

It can be seen that the WGAN-GP algorithm also reduces the FPR problem while obtaining higher TPR. The SMOTE algorithm is sensitive to noise and outliers, and if the selected minority class samples are surrounded by majority class samples, which may be noisy, the newly synthesized samples will overlap with most of the surrounding majority class samples, resulting in classification difficulties. While the unbalanced sample enhancement method is based on GAN through continuous training and iteration, the generator and discriminator compete with each other and enhance each other, and this mechanism of adversarial training makes GAN able to generate high-fidelity sample data.

### 3.2. Stroke Prediction

**Figure 3:**
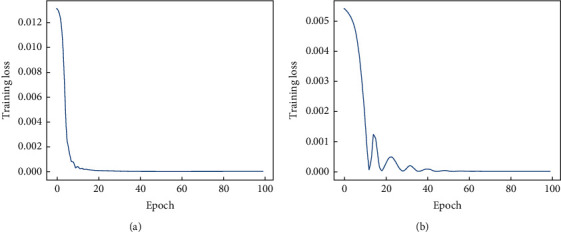
Training results of the proposed regression network. (a) The MSE loss of training data; (b) the MSE loss of validation data.

The stroke sample data set produced in this paper contains 10,000 sets of data, including 5,000 sets of positive and 5,000 sets of negative samples, and the positive samples are augmented using the WGAN-GP-based approach. The experimental data set is divided into training set, validation set, and test set in the ratio of 7 : 2 : 1 (1000 sets of test data are 500 sets of positive and negative samples, respectively, selected in the real sample set). The epoch is set to 100, and batch_size is set to 500 for training the regression model; the mean square error function is used as the loss function, and the optimizer is “Adam.” The training of the model stabilizes after 20 epochs, and the change curves of the training set loss function and the verification set loss function are shown in Figure 3.

From the results shown in Figure 3, it can be seen that the loss function of the regression model converges quickly on both the training and validation sets, which demonstrates the effectiveness of the proposed method. It should be noted that when the regression model is trained, the weights of its input parameters are calculated according to the correlation between the observed indicators and the stroke results. In this regard, we conducted statistics on the input parameters and the results of the diagnosis of stroke, and the analysis results showed that age, avg_glucose_level, hypertension, BMI, and heart_disease were more correlated with stroke. In fact, (1) the average age of stroke patients is much higher than the average age of those who do not suffer from stroke disease, and due to the decreased immunity of the elderly, the risk of suffering from various diseases will be higher; (2) the average blood glucose of stroke patients is higher, and the results of related studies have shown that diabetes is an independent risk factor for stroke [26], and hypertension is the most important and independent risk factor for stroke; (3) those with too high a BMI also have a higher probability of suffering from hypertension, and obesity will indirectly increase the probability of stroke; and (4) heart disease and other diseases will also increase the risk of stroke. Based on this, in this paper, the weight coefficients of five feature parameters, age, avg_glucose_level, hypertension, BMI, and heart_disease, are set to 1.0, while the weight parameters of the remaining five coefficients are set to 0.8.

**Figure 4:**
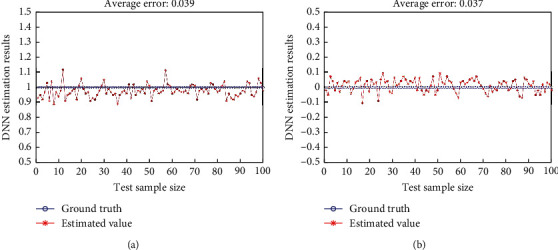
Prediction results of DNN. (a) Positive sample prediction results; (b) negative sample prediction results.

Further, 200 sets of data (100 sets each of positive and negative examples) were randomly selected from 1000 sets of test data, and the statistically predicted results are shown in Figure 4.

**Figure 5:**
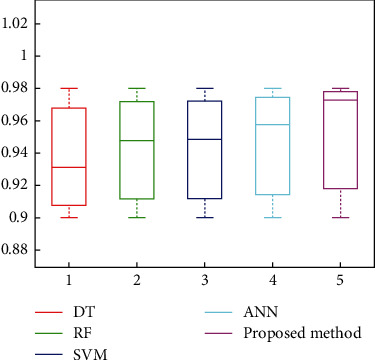
Comparison of prediction results of different algorithms.

From the statistical results, the average deviation of the prediction results for the positive sample is 0.039, and the maximum deviation is 0.116, while the average deviation of the prediction results for the negative sample is 0.037 and the maximum deviation is 0.103. Both the maximum deviation and the average deviation in the prediction results are small, which reflects the accuracy of the model prediction results. In this paper, we set the threshold value th = 0.9, i.e., the prediction result is judged to be correct when the relative deviation of the DNN output prediction value is within 10%. The accuracy of the positive and negative example samples in Figure 4 is 97% and 99%, respectively, and further statistically, the average accuracy of the positive and negative example samples is 97.6% and 98.2%, respectively, for all the test set samples. It can be seen that the model in this paper has high accuracy. In order to more objectively describe the effectiveness of the proposed model, we use the comprehensive indicator metric *F* to evaluate the prediction results (the higher the *F* score indicates the better the algorithm's prediction) [27], which is calculated by the following formula:

(8) $$\begin{array}{c} \begin{array}{c} P=\frac{\text{TP}}{\text{TP}+\text{FP}},\\ R=\frac{\text{TP}}{\text{TP}+\text{FN}},\\ F=\frac{2\text{P}\times \text{R}}{\text{P}+\text{R}}, \end{array} \end{array}$$

where TP denotes the prediction of a positive sample as having a stroke disease, FP denotes the prediction of a negative sample as having a stroke disease, FN denotes the prediction of a positive sample as having a nonstroke disease, *P* denotes the precision rate, and *R* denotes the recall rate. The proposed algorithm is compared with decision tree (DT) [28], random forest (RF) [29], support vector machine (SVM) [30], and artificial neural network (ANN) [31] classification algorithms, and the results of counting the *F* values on the test set are shown in Figure 5.

**Figure 6:**
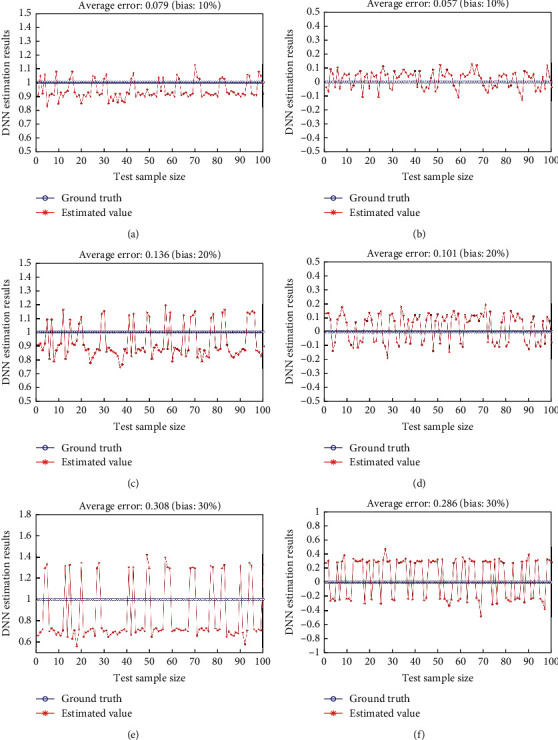
Prediction results of DNN with noise. (a) Prediction results for the positive sample with 10% deviation; (b) prediction results for the negative sample with 10% deviation; (c) prediction results for the positive sample with 20% deviation; (d) prediction results for the negative sample with 20% deviation; (e) prediction results for the positive sample with 30% deviation; (f) prediction results for the negative sample with 30% deviation.

It can be seen that compared with the commonly used machine learning classification algorithms DT, RF, SVM and ANN, the model in this paper is also optimal in terms of the comprehensive indicator metric *F* (the comprehensive indicator metric *F* of DT, RF, SVM, and ANN and the proposed algorithm are 0.9313, 0.9478, 0.9486, 0.9577, and 0.9728, respectively), indicating the effectiveness of the designed prediction model. To further validate the robustness of the method in this paper, we conducted ablation experiments with randomly selected 200 sets of test sample data *x*_1_, *x*_2_, *x*_3_, ⋯, *x*_10_ with set deviations of 10%, 20%, and 30%, and the prediction results of statistical DNN are shown in Figure 6.

As shown in Figure 6, as the deviation of the input observations increases, the value of the deviation from the predicted results for the positive and negative samples also increases. When the deviation of the input sample is set to 10%, the average accuracy of the positive and negative samples is 91% and 92%, respectively. When the deviation increases to 20%, the average accuracy of both positive and negative samples is lower than 60%, but the maximum deviation is only 0.247 and 0.188. However, when the input deviation is further increased to 30%, the magnitude of the positive and negative sample mean deviation increases to 0.308 and 0.286, respectively, and the accuracy of the prediction results is greatly reduced. It can be seen that when there is a small deviation in the input parameters of DNN, positive and negative samples can be effectively judged according to the output results of the network (there is a 10% bias in the input observations, and the maximum deviation in the prediction results for the positive and negative example samples is only 0.168 and 0.124), and the designed regression model has a certain robustness.

## 4. Discussion

The etiology of stroke is the dysfunction of brain cells caused by ischemia, hypoxia, degeneration, and necrosis due to cerebrovascular disease. The main cause of stroke is directly related to long-term hypertension, hyperlipidemia, and hyperglycemia without good treatment, which can lead to vascular atherosclerosis and plaque formation. In addition, cardiogenic emboli due to arrhythmias should not be ignored. Therefore, the model parameters in this paper are set up by assigning higher weights to the observations age, avg_glucose_level, hypertension, BMI, and heart_disease associated with these main causal factors in order to obtain more accurate estimates.

The data-driven deep learning method largely depends on the size of the sample size. It is difficult to collect a large number of positive stroke case data, and the serious imbalance between positive and negative case samples will greatly reduce the accuracy of predicting a few classes of samples. In this regard, we propose a deep learning network based on WGAN-GP to generate stroke-positive example data with high fidelity to achieve data augmentation, which in turn improves the generalization of the regression network. The Wasserstein distance used by WGAN-GP is differentiable and continuous, which makes its training process more stable, while the introduction of the gradient penalty mechanism can effectively control the characteristics of the generator's output data and obtain more diverse samples. The results of the data enhancement experiments show that WGAN-GP can effectively extract the key information of the stroke samples, and the generated stroke-positive example data is closer to the distribution of the real data.

On the basis of augmenting the positive example samples and optimizing the unbalanced stroke dataset, we designed a stroke prediction model based on regression networks. According to the general approximation theorem, the complex relationship between predicting individual stroke risk and characterizing individual potential stroke factors (such as *x*_1_, *x*_2_, *x*_3_, ⋯, *x*_10_) is modeled as an optimal solution problem for DNN. In fact, the risk factors of stroke are complex and changeable, and there is a complex mapping relationship between the predicted result *y* and the observed parameter *x*_1_, *x*_2_, *x*_3_, ⋯, *x*_10_, which is usually difficult to express by explicit function. We set up a regression network model to transform the medical diagnosis problem of stroke into a nonlinear mapping problem and use the powerful nonlinear fitting ability of the neural network to optimize the solution of the designed DNN. Experimental results on the test set demonstrate the effectiveness of the proposed model (in this paper, the average accuracy of positive and negative samples reached 97.6% and 98.2%, respectively, when th = 0.9 was set at a high decision threshold). Comparison experiments were conducted with DT, RF, and SVM classification algorithms, and further ablation experiment further verified the robustness of the proposed method.

In this paper, deep learning methods are applied to the field of medicine to provide a new way to prevent stroke disease. Preprocessing complex observation data associated with stroke disease and constructing stroke prediction models can assist doctors in diagnosis and reduce the influence of doctors' subjective factors on the diagnosis results. Effective preventive measures for high-risk individuals based on the prediction results are of great significance in reducing the incidence of stroke.

## 5. Conclusions

This paper presents a novel stroke prediction network model that was shown to be more competitive than current prediction methods. Moreover, this work presents an effective strategy for data augmentation in stroke-imbalanced samples. In this study, 10 factors such as avg_glucose_level, hypertension, and BMI were integrated, and a DNN-based stroke risk prediction model was built and verified on 1000 test samples. The average accuracy of positive and negative samples reached 97.6% and 98.2%, respectively.

Clinical prediction of the probability of stroke disease occurrence by a single influencing factor alone often lacks specificity, and assessment of multiple influencing factors in combination increases their specificity and improves the accuracy of prediction models. In this study, 10 factors, including avg_glucose_level, hypertension, and BMI, were integrated to construct a DNN-based stroke risk prediction model, which was validated on 1,000 sets of test samples, and the average accuracy of positive and negative case samples reached 97.6% and 98.2%, respectively. Further ablation experiments verified that the method in this paper has certain robustness. The test results show that the designed stroke prediction model has high application value, which can assist doctors in assessing and predicting stroke conditions and provide an objective basis for medical decisions.

Future work will focus on adapting the proposed stroke prediction model on observational data with missing characterizing attributes. This will pose a greater challenge to the generalizability of the designed stroke prediction network models. Additionally, collecting more stroke data further extends the sample set to better train deep learning models. Data enhancement and transfer learning are the main methods to deal with small sample learning problems. This paper studies the methods of data enhancement and obtains satisfactory results, but it requires personnel with domain knowledge to do a lot of data annotation work in the early stage. Further research on transfer learning methods, such as meta-learning and self-learning combined with attention mechanism, is needed in the future.

## Figures and Tables

**Table 1:** Characterization parameters and their quantitative values.

Classification of attribute features	Quantitative values for attribute features
Gender	Male =1; female = -1; others =0
Age	Real age
Hypertension	Yes =1; no = -1
Heart_disease	Yes =1; no = -1
Ever_married	Yes =1; no = -1
Work_type	Children = -2; govt_job = -1; never_worked =0; self-employed =1; private =2
Residence_type	Urban =1; rural = -1
Avg_glucose_level	Actual measured value
Bmi	Actual measured value
Smoking_status	Never smoked = -1; formerly smoked =1; smokes =2

**Table 2:** Details of the regression network.

Layer	Output_dim	Layer details
Hidden layer1	128	Input_dim: 10 Activation: Relu Dropout: 0.2
Hidden layer2	128	Activation: Relu Dropout: 0.2
Hidden layer3	256	Activation: Relu Dropout: 0.2
Hidden layer4	256	Activation: Relu Dropout: 0.0
Hidden layer5	512	Activation: Relu Dropout: 0.3
Hidden layer6	512	Activation: Relu Dropout: 0.3
Hidden layer7	1	—

**Table 3:** Comparison of augmentation results of different algorithms.

Algorithm	Index	
	TPR	FPR
WGAN-GP	0.9471	0.062
WGAN	0.9318	0.076
SMOTE	0.9105	0.091

## Data Availability

Previously reported data were used to support this study and are available at [https://www.kaggle.com/datasets/fedesoriano/stroke-prediction-dataset.]. These prior studies are cited at relevant places within the text as references [23].
